# Evaluation of the effect of intracanal cryotherapy on postoperative pain after retreatment: a randomized controlled clinical trial

**DOI:** 10.1007/s00784-025-06435-w

**Published:** 2025-06-25

**Authors:** Arzu Kaya Mumcu, Ecenur Tuzcu, Safa Kurnaz, Gülsen Kiraz

**Affiliations:** Department of Endodontics, Faculty of Dentistry, Kutahya Health Sciences University, Kutahya, 43100 Turkey

**Keywords:** Intracanal cryotherapy, Endodontic retreatment, Postoperative pain, Cold saline, VAS, Single-visit retreatment

## Abstract

**Objectives:**

This randomized controlled clinical trial aimed to evaluate the effect of intracanal cryotherapy using cold saline (2.5 °C) as a final irrigant on postoperative pain and analgesic consumption following single-visit nonsurgical endodontic retreatment of asymptomatic mandibular premolars.

**Materials and methods:**

Sixty systemically healthy patients with asymptomatic apical periodontitis in previously treated single-rooted mandibular premolars were enrolled and randomly assigned to two groups (*n* = 30 each). Both groups underwent standardized retreatment protocols, with the cryotherapy group receiving final irrigation with cold saline (2.5 °C) and the control group with room-temperature saline, both for 5 min. Postoperative pain was assessed using a 10-point Visual Analog Scale (VAS) at 6, 12, 24, 48, 72, and 168 h. Analgesic intake was also recorded. Statistical analysis was performed using the Chi-Square and Mann-Whitney tests with a significance level set at 0.05.

**Results:**

The cryotherapy group exhibited significantly lower pain scores at 6 h (*p* = 0.014) and 12 h (*p* = 0.028) postoperatively compared to the control group. No significant differences were observed at 24, 48, 72, or 168 h. Although fewer patients in the cryotherapy group reported analgesic use, this difference was not statistically significant (*p* = 0.278). No adverse events were reported.

**Conclusions:**

Intracanal cryotherapy using cold saline effectively reduced short-term postoperative pain following endodontic retreatment but did not significantly influence long-term pain or analgesic intake.

**Clinical relevance:**

Intracanal cryotherapy offers a simple, low-cost adjunct that may effectively reduce early postoperative pain and improve patient comfort in single-visit retreatment.

## Introduction

Primary root canal treatment (RCT) may sometimes fail to eliminate periapical pathology, necessitating non-surgical endodontic retreatment. This procedure can be performed in either a single or multiple visits. Single-visit retreatment is often preferred for its practicality, particularly in cases involving pulp necrosis, trauma, or the presence of a sinus tract, as it reduces the number of appointments and minimizes the risk of inter-appointment contamination [1]. However, this approach precludes the use of intracanal medicaments such as calcium hydroxide, which has demonstrated strong antimicrobial activity against resistant pathogens like Enterococcus faecalis and Candida albicans [2–4]. While multi-visit retreatment enhances disinfection, especially in anatomically complex canals, it is associated with extended treatment duration, increased cost, and potential weakening of dentin due to the effects of medicaments and the temporary nature of interim restorations [5–7]. Importantly, current evidence suggests that single- and multiple-visit retreatments produce comparable clinical outcomes in terms of postoperative pain and periapical healing [8]. Advances in endodontic techniques and materials further support the viability of single-visit retreatment as a reliable and effective option in appropriately selected cases [9].

Postoperative pain following retreatment remains a major concern, influencing both clinical outcomes and patient satisfaction [10]. During retreatment, despite careful instrumentation that does not exceed the apical foramen, the extrusion of dentinal debris, residual filling materials, irrigants, necrotic pulp tissue, and microorganisms into the periradicular tissues is frequently observed. This apical extrusion is associated with inflammation, postoperative pain, flare-ups, and delayed healing [11]. Notably, retreatment shows higher rates of pain and flare-ups compared to primary treatment, underscoring the importance of minimizing extrusion-related complications [12–15].

Effective postoperative pain management is crucial in endodontic therapy to enhance patient comfort and treatment outcomes. While NSAIDs, particularly ibuprofen, are commonly used for their proven analgesic effects, their use may lead to gastrointestinal, renal, and cardiovascular side effects [16, 17]. Consequently, interest in non-pharmacological and adjunctive strategies has increased. Approaches such as occlusal reduction [18, 19], low-level laser therapy [20], and irrigation activation [21] have shown varying degrees of success in managing postoperative discomfort.

Cryotherapy, the therapeutic application of cold, has long been used in medicine for its analgesic and anti-inflammatory effects through the reduction of tissue metabolism, blood flow, and nerve conduction velocity [22, 23]. In dentistry, it is typically applied post-surgically to reduce pain and swelling. Recently, intracanal cryotherapy—using cold saline for final irrigation—has gained attention in endodontics [24, 25]. Saline at 2.5 °C has been shown to lower root surface temperature, potentially inducing anti-inflammatory effects in periapical tissues [24, 25]. Its ability to reduce nerve conduction, hemorrhage, edema, and inflammation further supports its role in managing endodontic pain, mirroring its benefits in musculoskeletal conditions [26, 27].

Intracanal cryotherapy has been proposed as an adjunct for reducing postoperative discomfort; however, most evidence pertains to primary root canal treatments, with limited data on retreatment cases. A recent study found that while cryotherapy effectively reduces short-term pain, it does not influence long-term pain or analgesic use [28]. Although these results indicate potential benefits in retreatment, further evidence is needed. Therefore, this study aimed to evaluate the effect of intracanal cryotherapy on postoperative pain following single-visit, non-surgical root canal retreatment. The null hypothesis was that no significant difference would be observed between the cryotherapy and control groups in terms of postoperative pain and analgesic consumption.

## Materials and methods

This clinical study was approved by the Clinical Research Ethics Committee of Kutahya Health Sciences University (Approval No: 2023-07/03). The study protocol was registered in the ClinicalTrials.gov database with the identifier number NCT06764823. Comprehensive information about the study protocol, associated risks and benefits, and the voluntary nature of participation was provided to all candidates, and written informed consent was obtained prior to inclusion.

### Sample size calculation

The required sample size was calculated using G*Power v3.1.9.7 (Heinrich-Heine University, Düsseldorf, Germany), based on data from a previous study on postoperative pain [25]. Assuming an effect size of 0.8, a significance level of 0.05, and a power of 0.85, the analysis indicated that 30 patients per group were needed to detect a clinically significant difference. Thus, a total of 60 patients (30 per group) were included.

### Participant selection and eligibility criteria

This randomized clinical trial was conducted from July 2023 to July 2024 at the Department of Endodontics, Faculty of Dentistry, Kutahya Health Sciences University. Systemically healthy patients aged 18–59 years, who had not used analgesics or anti-inflammatories within 72 h, were eligible. Inclusion criteria comprised asymptomatic apical periodontitis in a single-rooted mandibular premolar previously treated with root canal therapy at least four years earlier. Asymptomatic apical periodontitis was defined as the presence of a persistent periapical radiolucency in a previously root canal-treated tooth, in the absence of clinical signs or symptoms. Clinical evaluations were performed by a calibrated endodontist following standardized diagnostic protocols. Teeth were confirmed to be free of spontaneous pain, tenderness to percussion or palpation, swelling, sinus tract and functional discomfort (assessed using a cotton roll bite test). Radiographic failure was defined as the presence of a periapical lesion with a periapical index (PAI) score of ≥ 4, measuring at least 2 × 2 mm in diameter. The quality of the root canal filling was classified based on length (2–4 mm short of apex) and homogeneity, defined as the absence of voids or visible gaps in the radiopaque filling mass. Only mandibular premolars with radiographically confirmed single-root morphology were included. Radiographic evaluations were performed using digital periapical radiographs (Planmeca ProX™, Helsinki, Finland) with a standardized exposure time (70 kVp, 8 mA, 0.2 s) and paralleling technique. Two periapical radiographs were obtained for each tooth at two horizontal angulations (orthoradial and 20° mesial) to assess root morphology, verify single-root anatomy, and exclude cases with suspected additional canals or ambiguous structures. During the retreatment procedure, the presence of a single canal was also confirmed clinically. All radiographs were assessed in a darkened room using an illuminated viewer and ×3.5 magnification. The evaluations were performed by a single calibrated endodontist (A.K.M), who had previously undergone a calibration session by examining 50 radiographs not included in the study. To minimize bias, the observer was blinded to treatment allocation.

Exclusion criteria included pregnancy or breastfeeding, preoperative pain, systemic conditions, recent antibiotic use (within 1 month), or analgesic use (within 72 h). Teeth with root fractures, canal calcifications, or internal/external resorption were also excluded. Only one tooth per participant was included. Informed consent was obtained from all participants.

### Randomization and intervention

Participants were randomly assigned to two groups using pre-prepared, sequentially numbered, opaque sealed envelopes to ensure allocation concealment. While patients were blinded to group allocation, the operator was not, due to the nature of the intervention. Allocation was revealed immediately before treatment.

Group 1 underwent conventional non-surgical retreatment followed by intracanal cryotherapy with 2.5 °C saline irrigation for 5 min. Group 2 received the same retreatment, followed by saline irrigation at room temperature. All treatments were performed by a single experienced endodontist (E.T.) under standardized clinical conditions.

### Root canal retreatment procedures

All retreatment procedures were performed in a single visit under standardized clinical conditions. Local anesthesia was administered using 1.8 mL of 4% articaine with 1:200,000 epinephrine (Ultracaine D-S Forte, İstanbul, Türkiye). After rubber dam isolation and removal of coronal restorations, access cavities were prepared with a round diamond bur under water cooling.

After identifying the root canal and the existing root filling material, removal of the previous filling was performed using ProTaper Universal Retreatment files (D1, D2, and D3; Dentsply Maillefer) in accordance with the manufacturer’s instructions, with an X-Smart IQ endodontic motor (Dentsply Sirona, Ballaigues, Switzerland). Canal negotiation was carried out using a size 15 K-file (Dentac, İstanbul, Türkiye) until the working length was reached, which was then confirmed radiographically with periapical radiographs. No chemical solvents were used during the removal of the root canal filling. To eliminate any residual filling material within the canal, a size 30 H-file (Dentac, İstanbul, Türkiye) was employed. Subsequently, root canal shaping was completed using the WaveOne Gold (Dentsply Maillefer, Ballaigues, Switzerland) file system with a size 45.05 instrument. During instrumentation, the canals were irrigated between each file using 2 mL of 2.5% sodium hypochlorite (NaOCl; Calasept, Nordiska Dental, Ängelholm, Sweden) delivered with side-vented needles (Dentac, İstanbul, Türkiye) positioned 2 mm short of the working length.

Following root canal preparation, final irrigation was performed using passive ultrasonic irrigation (PUI) with an IRRI S 21/25 tip (VDW, Munich, Germany) mounted on an ultrasonic device (VDW Ultra; VDW GmbH, Munich, Germany), set at 30% power. A total of 5 mL of 2.5% NaOCl was activated in three 20-second cycles, with the tip positioned 2 mm short of the working length, avoiding canal wall contact. This was followed by 5 mL saline irrigation using side-vented NaviTip^®^ needles, and activation of 2 mL 17% EDTA (Saver, Imicryl, Türkiye) for 1 min using the same ultrasonic protocol. In the cryotherapy group (*n* = 30), canals were irrigated with 5 mL of 0.9% saline at 2.5 °C for 5 min. In the control group (*n* = 30), 5 mL of room-temperature saline was used for the same duration. The saline solution used for cryotherapy was stored in a laboratory-grade calibrated refrigerator at 2.5 °C for at least 2 h prior to the procedure. Immediately before use, the temperature of the solution was verified using a digital thermometer to ensure accuracy. During irrigation, the saline was continuously delivered with a syringe pre-cooled to the same temperature, and the irrigation was completed within 5 min to minimize thermal deviation. The solution was refreshed if any delay occurred to prevent temperature rise. These steps were performed to maintain the irrigant at a constant temperature of 2.5 °C throughout the procedure [24, 25]. All canals were then dried with sterile paper points and obturated using WaveOne Gold gutta-percha and AH Plus sealer (Dentsply/De Trey, Konstanz, Germany). Access cavities were restored with composite resin (Clearfil, Kuraray, Japan), and occlusion was checked and adjusted.

All patients (*n* = 60) were instructed to complete a standardized pain diary using a 10-point visual analog scale (VAS). The scale was explained prior to treatment to ensure accurate reporting. Pain scores were recorded at 6, 12, and 24 h, and on days 2, 3, and 7 postoperatively. Analgesic intake, including timing and dosage, was also documented. Patients who used analgesics during follow-up were excluded from the final analysis. Completed diaries were returned in person, electronically, or via telephone reporting. Demographic and treatment data were collected for all participants.

### Statistical analysis

Data were analyzed using the SPSS software (version 26.0; SPSS Inc., Chicago, IL). The normality of the data distribution was assessed using the Shapiro-Wilk test. The Mann-Whitney U test was used for comparing non-parametric data between groups. The Chi-square test was applied for categorical data. Descriptive statistics (mean, standard deviation, minimum, and maximum) were presented for comparisons between groups. A p-value of less than 0.05 was considered statistically significant.

## Results

**Fig. 1:**
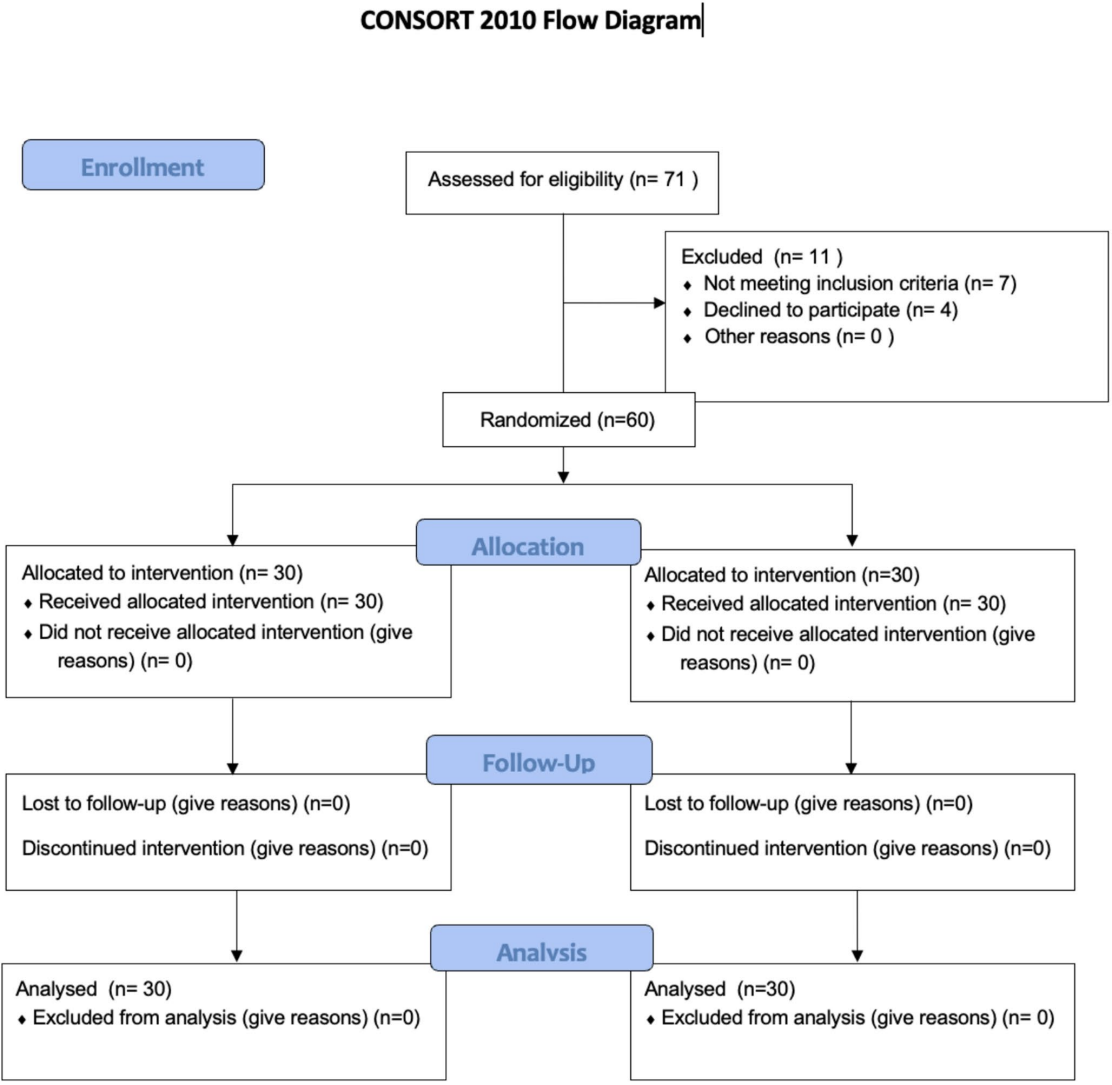
CONSORT flow diagram

This study was conducted in accordance with the CONSORT guidelines, and a summary of the study is presented in the CONSORT flow diagram (Fig. 1). A total of 71 patients who applied to the Department of Endodontics at Kutahya Health Sciences University Faculty of Dentistry for retreatment and met the inclusion criteria were enrolled in the study. However, four patients were excluded: seven patients not meeting the inclusion criteria, and four patients declined to participate. The remaining participants were randomly assigned into two groups.

All patients attended their follow-up appointments. Demographic data regarding age and gender are presented in Table 1. No statistically significant difference was found between the groups in terms of age and gender (*P* > 0.05).

**Table 1:** Demographic data according to groups

Variable		Control	Cryotherapy	*P* value
Age(years)	Mean±SD*	38.00 ± 12.315	36.43 ± 11.702	0.615
	Range	19–61	19–55	
Sex	Females	17 (56.7%)	12 (40%)	0.196
	Males	13 (43.3%)	18 (60.0%)	

*Standart deviation

Table 2 presents the mean and standard deviation values of pain levels for both the cryotherapy and control groups. At the 6th and 12th hours, pain levels in the cryotherapy group were lower than those in the control group, and the difference was statistically significant (*P* < 0.05). However, at 24, 48, and 72 h, as well as at the one-week mark, there were no statistically significant differences in pain levels between the two groups (*P* > 0.05).

**Table 2:** Means of VAS before and after treatment in two study arms

Study Arms	Hours after treatment					
	6 h	12 h	24 h	48 h	72 h	168 h
Control	3.43±2.979	2.67 ± 2.682	1.70 ± 2.087	1.27 ± 1.799	0.73 ± 1.337	0.37 ± 0.765
Cryotherapy	1.67 ± 2.057	1.07 ± 1.363	0.80 ± 1.270	0.47 ± 1.008	0.33 ± 0.802	0.13 ± 0.434
*p- value*	0.014*	0.028*	0.096	0.063	0.198	0.161

*p < 0,05 statistical difference

Regarding analgesic consumption, 3 patients in the cryotherapy group and 6 patients in the control group reported using analgesics. There was no statistically significant difference between the groups in terms of analgesic intake. Most analgesic use was reported within the first 48 h following the procedure (Table 3).

**Table 3:** Analgesic consumption after treatment

Study arms	6 h		12 h		24 h		48 h		72 h		168 h		Total analgesic consumption
	Yes	No	Yes	No	Yes	No	Yes	No	Yes	No	Yes	No	
Control	2	28	2	28	1	29	1	29	0	30	0	30	6
Cryotherapy	1	29	1	29	1	29	0	30	0	30	0	30	3
*p* value													0.278

*p < 0.05 statistical difference

## Discussion

This study aimed to evaluate the effect of intracanal cryotherapy (sterile saline at 2.5 °C) applied after retreatment on postoperative pain and analgesic consumption at different time intervals (6, 12, 24, 48, and 72 h, and 1 week). Pain levels in the cryotherapy group were found to be significantly lower than those in the control group at the 6th and 12th hours. However, no statistically significant differences were observed between the groups at the subsequent time points. Similarly, there was no statistically significant difference between the groups in terms of analgesic use. Therefore, the null hypothesis of this study was partially rejected.

Postoperative pain, typically resulting from periradicular inflammation, is a common and undesirable outcome of root canal treatment for both clinicians and patients [9, 29]. The reported incidence of postoperative pain following endodontic retreatment varies widely, ranging from 3 to 58% of patients [29]. This pain may stem from biological irritants, such as microorganisms, or from chemical and mechanical stimuli introduced during treatment [30]. Endodontic procedures may trigger the release of inflammatory mediators—such as prostaglandins, leukotrienes, bradykinin, substance P, and platelet-activating factor—into periapical tissues [31]. Some mediators, notably bradykinin and prostaglandins, directly stimulate or sensitize nociceptors, intensifying pain perception [32]. The resulting periradicular inflammation induces vasodilation and increased vascular permeability, leading to edema and an exaggerated tissue response [31].

Pain management following root canal treatment is commonly achieved through the use of medications such as acetaminophen, antihistamines, steroidal and non-steroidal anti-inflammatory drugs (NSAIDs), salicylic acid, narcotics, intracanal medicaments, or long-acting anesthetics [33, 34]. However, due to the wide range of side effects associated with NSAIDs and existing limitations regarding their use for managing post-endodontic pain, non-pharmacological interventions—such as cryotherapy—have gained increasing popularity in recent years [35].

Cryotherapy is widely used extraorally after surgical procedures, and its intracanal application in endodontics was first introduced by Vera et al. [24]. They reported that 2.5 °C saline reduced the external root surface temperature by approximately 10 °C—sufficient to elicit local anti-inflammatory effects by slowing the inflammatory response, decreasing pain mediator release, and minimizing periapical edema. Although there is no consensus on the optimal protocol, most studies have applied 20 mL of 2.5 °C saline for 5 min [25, 27, 36, 37]. In line with these studies, our protocol also utilized 20 mL of cold saline at 2.5 °C for 5 min as the final irrigant.

Most cryotherapy studies have used the EndoVac negative apical pressure system (Kerr Endo, USA) to prevent vapor lock, ensure continuous cold irrigant delivery to the apical third, and minimize apical extrusion, thereby reducing periapical inflammation [24, 38–40]. However, due to its limited availability and a recent systematic review showing no significant difference between EndoVac and conventional needle irrigation [40], the present study employed conventional irrigation. A 31-gauge NaviTip^®^ side-vented needle was positioned 2 mm short of the working length, following prior recommendations to minimize extrusion and ensure safety [41, 42]. Even with this conventional approach, cryotherapy effectively reduced postoperative pain compared to the control group, consistent with findings by Keskin et al. [25].

The prognosis of endodontically retreated teeth may differ from primary treatments due to factors such as altered microbial profiles—often involving resistant species like *Enterococcus faecalis*—and the presence of residual filling materials that impede disinfection [43–46]. Therefore, effective canal disinfection is critical, especially in single-visit retreatments. Karaoğlan et al. [47], reported that treatment success in asymptomatic, single-rooted teeth was independent of the number of visits, highlighting the importance of adequate disinfection [48]. To enhance irrigant efficacy in challenging areas, activation techniques such as passive ultrasonic irrigation (PUI) are recommended, with ultrasonic activation being the most commonly employed method [48, 49]. Studies show that placing the ultrasonic tip 1–3 mm short of the working length does not significantly affect debris extrusion or postoperative pain compared to conventional needle irrigation [50–52] In this study, PUI was used with the tip positioned 2 mm short of the working length to optimize disinfection while minimizing postoperative pain.

The hybrid instrumentation protocol involving the sequential use of PTUR and WOG files, while introducing potential variability, reflects realistic clinical scenarios. Although PTUR files are effective in removing root filling materials, additional shaping is often necessary in the apical region. Owing to its reciprocating motion, WOG offers superior shaping capability and increased resistance to cyclic fatigue compared to continuous rotary systems. Therefore, in our study, PTUR was employed for filling removal, whereas WOG was used for subsequent canal shaping. Moreover, a recent study by Harorlı et al. [53]. reported that the PTUR + WOG combination resulted in significantly less apical debris extrusion and shorter retreatment time compared to the Reciproc + OneReci (RE + OR) combination, particularly in non-perforated teeth. These findings suggest that the sequential use of rotary and reciprocating systems can enhance both clinical safety and procedural efficiency. Although our study did not directly assess debris extrusion, minimizing this phenomenon is critically important to prevent postoperative pain and inflammation. Therefore, we believe that the instrumentation protocol adopted in our study is not only consistent with current literature but also representative of scenarios frequently encountered in clinical endodontic practice.

Various methods have been employed to measure and assess postoperative pain, with the Visual Analog Scale (VAS) being one of the most commonly used tools in endodontic research due to its simplicity, reliability, and validity when properly administered [27, 51, 54]. The VAS is easy for patients to understand and has been widely adopted in studies evaluating postoperative pain in endodontics. Accordingly, the present study used a 0–10 VAS, where 0 indicates no pain and 10 represents the most severe imaginable pain. A key challenge in pain assessment is the inherently subjective nature of pain perception and reliance on patient self-reporting [55]. Therefore, the design of the assessment tool is critical—it must be clearly comprehensible to patients and easily interpreted by researchers [56]. To ensure accurate and consistent pain reporting, the VAS was thoroughly explained to all participants prior to data collection.

In this study, pain levels at the 6th and 12th hours were significantly lower in the cryotherapy group compared to the control group. Similar findings have been reported in studies on primary root canal treatments, where cryotherapy consistently reduced postoperative pain [25, 27, 36, 57]. Ghabraei et al. [28], also observed significantly lower pain at the 6th hour in cryotherapy cases following single-visit retreatment. While their results align with ours, the additional reduction at the 12th hour in our study may be attributed to the use of ultrasonic irrigation activation, which was not part of their protocol. Supporting this, previous research has shown that passive ultrasonic irrigation leads to lower postoperative pain compared to conventional needle irrigation [52].

In the present study, there was no statistically significant difference between the cryotherapy and control groups in terms of analgesic consumption. These findings are consistent with the results reported by Ghabraei et al. [28], who found that intracanal cryotherapy in single-visit retreatment cases was effective in reducing short-term postoperative pain, but did not significantly affect long-term postoperative pain or analgesic intake. Therefore, the findings of our study support the notion that intracanal cryotherapy may be considered a non-pharmacological adjunct for reducing short-term postoperative pain following retreatment procedures.

Postoperative pain is influenced by multiple factors, including age, gender, tooth type, apical foramen size, and preoperative periapical lesion size, as well as various unknown variables [15, 58, 59]. To reduce their impact, only systemically healthy, asymptomatic patients were included, following the criteria of Keskin et al. [25]. As preoperative pain is a known predictor of postoperative discomfort [58]. this selection helped minimize variability. Patients were randomly allocated to groups, with no significant differences in age or gender between the cryotherapy and control groups. To standardize conditions, only single-rooted mandibular premolars with a periapical index (PAI) score of 4 were treated, reducing anatomical variability [27, 36, 57]. All procedures were completed in a single visit to eliminate the influence of intracanal medicaments [25, 60, 61]. To control for operator-related variability, all treatments were performed by the same endodontist (E.T.), who had three years of clinical experience and followed a standardized protocol.

Although both single-visit and multiple-visit protocols are commonly employed in root canal retreatment, there is still no clear consensus regarding their relative effectiveness. Single-visit retreatment offers practical advantages, such as fewer appointments, reduced risk of inter-appointment contamination, and improved patient compliance [1, 62]. However, its primary limitation is the inability to perform inter-appointment disinfection using intracanal medicaments, such as calcium hydroxide, which possess antimicrobial activity against resistant microorganisms like Enterococcus faecalis and Candida albicans [2–4]. Even so, these medicaments may not always effectively eliminate microorganisms residing in anatomically complex areas such as dentinal tubules, isthmuses, or lateral canals [5], and prolonged exposure to them may weaken dentin and increase the risk of fracture [6]. While multiple-visit retreatments allow for extended disinfection, there is no compelling evidence indicating that root canal retreatment in uncomplicated cases must necessarily be performed in multiple visits, and current literature does not clearly favor one protocol over the other [63]. A prospective clinical study reported no significant difference in success rates between single-visit and two-visit retreatments in single-rooted teeth with asymptomatic apical periodontitis, suggesting that the number of visits may not be a determining factor in treatment outcomes [47]. Instead, effective cleaning, shaping, and canal disinfection remain the most critical elements for success [43, 64–66]. In the present study, a single-visit protocol was selected to eliminate potential confounding factors such as the influence of intracanal medicaments or leakage from temporary restorations between appointments, in order to more directly assess the isolated effect of cryotherapy on postoperative pain.

Studies on postoperative pain are inherently limited by variations in study design, preoperative tooth conditions, treatment protocols, pain definitions, and the subjective nature of pain assessment and data collection [67]. A key challenge is the reliance on patient-reported outcomes, as pain cannot be objectively measured [55]. Therefore, the design of the pain assessment tool is critical—it must be easily understood by patients and reliably interpreted by researchers. In this study, the protocol was clearly explained to all participants to ensure consistent reporting. Operator blinding was not feasible due to the perceptible temperature of the irrigant; however, both patients and the statistician were blinded to group allocation to minimize bias. Additionally, the findings cannot be generalized to other tooth types. Further research with larger samples is needed, particularly in symptomatic cases, to assess long-term outcomes and biological effects, including inflammatory biomarkers. One limitation of the present study is that the volume and extent of the pre-existing root canal filling were not quantitatively assessed. Although the homogeneity and radiographic density of the fillings were qualitatively evaluated to maintain consistency, the absence of volumetric analysis may limit the ability to fully interpret the relationship between the preoperative filling characteristics and the extent of debris extrusion during retreatment. Another limitation is that all radiographic assessments were performed using two-dimensional periapical radiographs, which inherently lack the ability to capture three-dimensional anatomical variations. Despite the use of multiple angulations and standardized evaluation protocols, subtle morphological details such as isthmuses or lateral canals may have been overlooked. Future studies utilizing cone-beam computed tomography (CBCT) may provide a more accurate assessment of canal anatomy and preoperative conditions.

## Conclusion

Despite its limitations, this study demonstrates that intracanal cryotherapy significantly reduces short-term postoperative pain in asymptomatic retreatment cases, offering a clinically valuable, non-pharmacological alternative for pain management. Although it did not influence long-term pain or analgesic consumption, its simplicity, affordability, and absence of systemic side effects make it a promising adjunctive technique in single-visit retreatment protocols.

## Data Availability

No datasets were generated or analysed during the current study.
